# Pregnancy, Birth, Infant, and Early Childhood Neurodevelopmental Outcomes among a Cohort of Women with Symptoms of Zika Virus Disease during Pregnancy in Three Surveillance Sites, Project *Vigilancia de Embarazadas con Zika* (VEZ), Colombia, 2016–2018

**DOI:** 10.3390/tropicalmed6040183

**Published:** 2021-10-12

**Authors:** Marcela Mercado-Reyes, Suzanne M. Gilboa, Diana Valencia, Marcela Daza, Van T. Tong, Romeo R. Galang, Christina M. Winfield, Shana Godfred-Cato, Mónica Benavides, Julie M. Villanueva, Jennifer D. Thomas, Jonathan Daniels, Sherif Zaki, Sarah Reagan-Steiner, Julu Bhatnagar, Jarad Schiffer, Evelene Steward-Clark, Jessica N. Ricaldi, Johana Osorio, Christina L. Sancken, Lissethe Pardo, Sarah C. Tinker, Kayla N. Anderson, Angelica Rico, Veronica K. Burkel, Jacob Hojnacki, Miranda J. Delahoy, Maritza González, May B. Osorio, Cynthia A. Moore, Margaret A. Honein, Martha Lucia Ospina Martinez

**Affiliations:** 1 Instituto Nacional de Salud, Bogotá 111321, Colombia; mmercado@ins.gov.co (M.M.-R.); mdaza@ins.gov.co (M.D.); mbenavides@ins.gov.co (M.B.); lpardo@ins.gov.co (L.P.); arico@ins.gov.co (A.R.); magonzalez@ins.gov.co (M.G.); mosorio@ins.gov.co (M.B.O.); mospina@ins.gov.co (M.L.O.M.); 2 National Center on Birth Defects and Developmental Disabilities, Centers for Disease Control and Prevention, Atlanta, GA 30329, USA; ile9@cdc.gov (D.V.); vct2@cdc.gov (V.T.T.); kwf7@cdc.gov (C.M.W.); nzt6@cdc.gov (S.G.-C.); yof5@cdc.gov (C.L.S.); zzu9@cdc.gov (S.C.T.); lxx7@cdc.gov (K.N.A.); cam0@cdc.gov (C.A.M.); mrh7@cdc.gov (M.A.H.); 3 Research Division, Vysnova Partners, Landover, MD 20785, USA; joanaoup@gmail.com; 4 National Center for Chronic Disease Prevention and Health Promotion, Centers for Disease Control and Prevention, Atlanta, GA 30329, USA; ydh0@cdc.gov; 5 National Center for Emerging and Zoonotic Infectious Diseases, Centers for Disease Control and Prevention, Atlanta, GA 30329, USA; jfv3@cdc.gov (J.M.V.); fsu8@cdc.gov (J.D.T.); yer1@cdc.gov (J.D.); sxz1@cdc.gov (S.Z.); sor1@cdc.gov (S.R.-S.); zrn1@cdc.gov (J.B.); 6 National Center for Immunization and Respiratory Diseases, Centers for Disease Control and Prevention, Atlanta, GA 30329, USA; aku3@cdc.gov (J.S.); eqs2@cdc.gov (E.S.-C.); 7 National Center for HIV/AIDS, Viral Hepatitis, STD, and TB Prevention, Centers for Disease Control and Prevention, Atlanta, GA 30329, USA; mpi7@cdc.gov; 8 Eagle Medical Services, LLC, San Antonio, TX 78248, USA; xee5@cdc.gov; 9 Oak Ridge Institute for Science and Education, Oak Ridge, TN 37830, USA; jhojnacki100@gmail.com; 10 Eagle Global Scientific, LLC, San Antonio, TX 78248, USA; vuo0@cdc.gov

**Keywords:** Zika virus, pregnancy, Colombia, birth defects, neurodevelopment

## Abstract

Project *Vigilancia de Embarazadas con Zika* (VEZ), an intensified surveillance of pregnant women with symptoms of the Zika virus disease (ZVD) in Colombia, aimed to evaluate the relationship between symptoms of ZVD during pregnancy and adverse pregnancy, birth, and infant outcomes and early childhood neurodevelopmental outcomes. During May–November 2016, pregnant women in three Colombian cities who were reported with symptoms of ZVD to the national surveillance system, or with symptoms of ZVD visiting participating clinics, were enrolled in Project VEZ. Data from maternal and pediatric (up to two years of age) medical records were abstracted. Available maternal specimens were tested for the presence of the Zika virus ribonucleic acid and/or anti-Zika virus immunoglobulin antibodies. Of 1213 enrolled pregnant women with symptoms of ZVD, 1180 had a known pregnancy outcome. Results of the Zika virus laboratory testing were available for 569 (48.2%) pregnancies with a known pregnancy outcome though testing timing varied and was often distal to the timing of symptoms; 254 (21.5% of the whole cohort; 44.6% of those with testing results) were confirmed or presumptive positive for the Zika virus infection. Of pregnancies with a known outcome, 50 (4.2%) fetuses/infants had Zika-associated brain or eye defects, which included microcephaly at birth. Early childhood adverse neurodevelopmental outcomes were more common among those with Zika-associated birth defects than among those without and more common among those with laboratory evidence of a Zika virus infection compared with the full cohort. The proportion of fetuses/infants with any Zika-associated brain or eye defect was consistent with the proportion seen in other studies. Enhancements to Colombia’s existing national surveillance enabled the assessment of adverse outcomes associated with ZVD in pregnancy.

## 1. Introduction

The Zika virus is a flavivirus transmitted to humans primarily by the bite of infected mosquitoes of the *Aedes Stegomyia* species [1] and can cause fever, rash, and joint pain in infected individuals. Sexual and transplacental transmission of the Zika virus has been demonstrated [2,3]. A congenital Zika virus infection can cause microcephaly and severe birth defects of the brain and eye [4,5,6,7] and has been associated with neurodevelopmental abnormalities in the child, such as seizures, joint contractures, swallowing difficulties, vision impairments, hearing loss, and delayed achievement of developmental milestones [4,8,9,10,11,12,13,14,15,16,17,18,19,20,21]. Most of these neurodevelopmental abnormalities have been observed in children with Zika-associated birth defects of the brain or eye, although there have been some neurodevelopmental abnormalities in children who are phenotypically unaffected at birth [11,22,23].

Colombia began national surveillance of the symptomatic Zika virus disease (ZVD) in August 2015 through its public health surveillance platform, *Sistema de Vigilancia en Salud Pública* (Sivigila). The Zika virus outbreak peaked in Colombia in February 2016, coinciding with the request by the government of Colombia for technical assistance from the United States Centers for Disease Control and Prevention (CDC) to strengthen Colombia’s ongoing surveillance of symptomatic ZVD in pregnant women. Colombia declared an end to the outbreak in July 2016, with over 100,000 cases of symptomatic ZVD reported to Sivigila. Among them were 18,117 pregnant women; one-third of symptomatic ZVD cases in pregnant women in Colombia were laboratory-confirmed with the Zika virus infection [24].

CDC supported the national public health institution (*Instituto Nacional de Salud*, INS)-led Project *Vigilancia de Embarazadas con Zika* (VEZ), which was an intensified surveillance of pregnant women with symptomatic ZVD and their children at clinical sites in three cities in Colombia—Barranquilla, Cúcuta, and Cali. These sites were selected because of their high burden of symptomatic ZVD at the beginning of the outbreak. The aim of this first analysis of data from Project VEZ is to evaluate the relationship between ZVD during pregnancy and a spectrum of adverse pregnancy, birth, and infant outcomes and early childhood neurodevelopmental outcomes, considering the timing of symptom onset during pregnancy and the presence of laboratory evidence of confirmed or presumptive Zika virus infection during pregnancy.

## 2. Materials and Methods

### 2.1. Eligibility Criteria and Medical Records Abstraction

Women were enrolled in Project VEZ if they were (a) currently pregnant, (b) receiving prenatal care or delivering at the selected clinical sites within the three cities, and (c) either experiencing two or more ZVD-compatible symptoms at enrollment or reported two or more ZVD-compatible symptoms since their last menstrual period (LMP). Alternatively, women could be identified through Sivigila with laboratory-confirmed or clinically compatible ZVD since LMP and were enrolled if they received prenatal care or delivered at the selected clinical sites. Enrollment took place between May–November 2016. After enrollment, a trained field staff abstracted data from prenatal care (including ultrasound reports), delivery hospitalization, and infant records with standardized data collection forms. Pediatric follow-up data from medical records were abstracted on the children if available until they reached up to two years of age.

Field staff captured additional pediatric follow-up data through six health brigades (two in each of the three cities) conducted by INS between April–August 2018 to conduct screenings and evaluations based on the nationally recommended guidelines for the assessment of children born to mothers with symptoms of ZVD during pregnancy. Assessments included a general pediatric and neuropediatric evaluation, a hearing screen using auditory brain stem responses, an ophthalmological exam assessing external structures of the eye as well as eye movement and alignment, and a fundus examination through indirect ophthalmoscopy, and developmental milestone screenings. Mothers were asked to bring the child’s medical record to the brigade; any information relevant to Project VEZ that was either in the child’s record or captured during the clinical encounters that were part of the brigade was abstracted by field staff onto the standardized data collection forms.

### 2.2. Specimen Collection and Testing

Specimen collection and Zika virus testing were based on Colombian national guidelines [25]. For all VEZ participants, maternal serum and urine specimens were to be collected as part of the protocol, at enrollment, during pregnancy as part of routine clinical care, and at delivery. If amniotic fluid was collected for clinical reasons other than Zika, then an aliquot was taken for Zika virus testing. At delivery, umbilical cord blood and tissue samples of the placenta and umbilical cord were collected but no additional specimens were collected from live-born infants. For spontaneous or induced pregnancy losses, fetal tissue was collected when possible. For VEZ participants originally identified through national surveillance (Sivigila), a blood sample was collected as soon as possible after symptom onset.

Laboratory procedures for detecting possible Zika virus infections evolved over time as assays and training became available in Colombia. For maternal serum samples that were collected as part of Sivigila, INS protocol was to collect and test serum samples within five days of symptom onset using one of two nucleic acid amplification testing (NAAT) procedures to detect viral ribonucleic acid (RNA)—a single target real-time reverse transcription polymerase chain reaction (rRT-PCR) assay (singleplex assay), based on a published method with reflex testing for dengue and chikungunya for those with negative Zika rRT-PCR results [26] or the Trioplex rRT-PCR assay, which detects RNA from all three viruses simultaneously [27,28] depending on when the case occurred during the outbreak. Zika virus IgM antibody testing was not performed at INS on samples collected as part of Sivigila.

All specimens collected as part of Project VEZ were sent to INS for processing in its virology or pathology laboratories; aliquots were immediately prepared and stored at −80 °C. It was not feasible to limit collection and testing to within five days of symptom onset for these specimens. INS laboratory scientists performed NAAT on a subset of 50 maternal serum, urine, and tissue samples using the singleplex assay. One aliquot of all available Project VEZ samples was subsequently sent to CDC. INS also created a biorepository of samples related to the Zika virus outbreak in Colombia, in which all remaining samples from Project VEZ are stored.

CDC laboratory scientists tested all available specimens of maternal serum and maternal urine for the presence of Zika viral RNA using the Trioplex assay. Frozen placental, umbilical cord, and fetal tissues were thawed, fixed in 10% neutral buffered formalin, and then embedded in paraffin. CDC laboratory scientists also tested fixed tissues for the presence of Zika viral RNA using a conventional Zika virus RT-PCR, followed by Sanger sequencing of the PCR products [29]. The search for homologies to known sequences was performed by using the BLAST nucleotide database (http://blast.ncbi.nlm.nih.gov/Blast.cgi, accessed on 18 October 2018). Serologic testing of all maternal serum samples to detect the Zika virus IgM antibodies was performed using the Zika virus MAC-ELISA (CDC, Atlanta, GA, USA) [26,30]. Dengue IgM testing was performed for those positive by Zika IgM using the InBios DENV Detect IgM Capture ELISA (InBios, Seattle, WA, USA).

Additionally, any results of Zika virus laboratory testing conducted during pregnancy found in the medical records of participants in Project VEZ were abstracted.

### 2.3. Laboratory Evidence of Zika Virus Infection during Pregnancy

Using all available laboratory results (i.e., Sivigila results, results abstracted from medical records, results from INS laboratories, and results from CDC laboratories), an algorithm to define laboratory evidence of Zika virus infection during pregnancy was developed by the Colombian INS in consultation with CDC (Table S1).

### 2.4. Zika-Associated Birth Defects and Other Pregnancy Outcomes

Project VEZ applied the updated CDC surveillance case definition of Zika-associated birth defects: brain abnormalities with or without microcephaly and structural eye abnormalities. Infants with early brain malformations such as neural tube defects and holoprosencephaly were excluded in accordance with the case definition [31]. Microcephaly at birth was defined as head circumference <3rd percentile for gestational age and sex by INTERGROWTH-21st standards for measurements collected ≤2 weeks after birth [32]. Postnatal-onset microcephaly was defined for children with no evidence of microcephaly at birth but who had head circumference measurements collected >2 weeks after birth <3rd percentile for the child’s sex and age based on the World Health Organization child growth standards [33] or a downward trajectory of head circumference percentiles with the most recent measurement <3rd percentile. Age at measurement was adjusted for gestational age in infants born at <40 weeks’ gestational age through 24 months’ chronological age. A clinical diagnosis of microcephaly or mention of microcephaly or small head size in the medical record was neither necessary nor sufficient to meet the case definition. Results from a prenatal ultrasound, pathology examination (in cases of pregnancy loss), and postnatal neuroimaging (in cases of live birth) were reviewed for the presence of brain abnormalities. Results of ophthalmologic examination were reviewed for the presence of eye defects.

Other pregnancy outcomes assessed were pregnancy loss <20 weeks’ gestation and pregnancy loss ≥20 weeks’ gestation. Among singleton, liveborn infants without Zika-associated birth defects, we also assessed preterm delivery (<37 weeks’ gestation), low birth weight (<2500 g), and small for gestational age (<10th percentile for birth weight by gestational age and sex using INTERGROWTH-21st standards). Gestational age at delivery was assigned based on a hierarchy; the estimated date of delivery based on first trimester ultrasound data was prioritized. If first trimester ultrasound data were unavailable, dating based on second trimester ultrasound, clinician estimate at delivery, or reported date of last menstrual period, was used. We also assessed deaths among all live-born infants in the first year of life.

### 2.5. Early Childhood Neurodevelopmental Outcomes 

The definition of follow-up was the presence of clinical information in the medical record after two weeks post-birth. Among this group of children, we explored early childhood neurodevelopmental outcomes. Neurodevelopmental outcomes were categorized as the following neurodevelopmental sequelae documented in the medical record or clinical notes at a health brigade: seizures, swallowing abnormalities, tone abnormalities, movement abnormalities, arthrogryposis, visual impairment, or hearing abnormalities, or potential delay in achieving developmental milestones. Potential delay in achieving developmental milestones was based on documentation of developmental “alert scores” or areas of concern requiring further assessment, based on the Colombian national, standardized, validated developmental screening assessment normed on a diverse sample of Colombian children, the *Escala Abreviada de Desarrollo* (EAD-1) [34]. The EAD-1 assesses development in four domains—gross motor, fine motor, personal–social, and hearing and language. Based on a scaled score, a child could be classified as having an alert score in any of the domains.

### 2.6. Data Entry and Analysis

All abstracted data were entered into INS/CDC-developed forms in REDCap Version 6 (Research Electronic Data Capture; Version 6; Nashville, TN, USA). We calculated the proportion of adverse pregnancy outcomes and Zika-associated birth defects among completed pregnancies with a known outcome, as well as in the subgroup of completed pregnancies with a confirmed or presumptive Zika virus infection. We also stratified the analyses of adverse pregnancy outcomes and Zika-associated birth defects by trimester of ZVD symptom onset, including periconceptional ZVD symptom onset (defined as up to 56 days before the estimated date of conception). In the cohort of children with data abstracted after two weeks post-birth, we calculated the proportion with neurodevelopmental sequelae and the proportion of children with EAD-1 alert scores by domain separately among the children with Zika-associated birth defects and those without. Chi-square testing with the significance level at *p* < 0.05 was used to assess differences in proportions. For statistical testing of the differences in Zika-associated birth defects by trimester of ZVD symptom onset, we compared the first and second trimesters only due to limited sample sizes during the periconceptional period and third trimesters; for other adverse pregnancy outcomes, we compared across the three trimesters. When the expected cell size was less than five, Fischer’s exact testing with significance level at *p* < 0.05 was used. All data analyses were conducted in SAS 9.4 (SAS Institute, Cary, NC, USA).

## 3. Results

### 3.1. Characteristics of the Study Population

**Figure 1:**
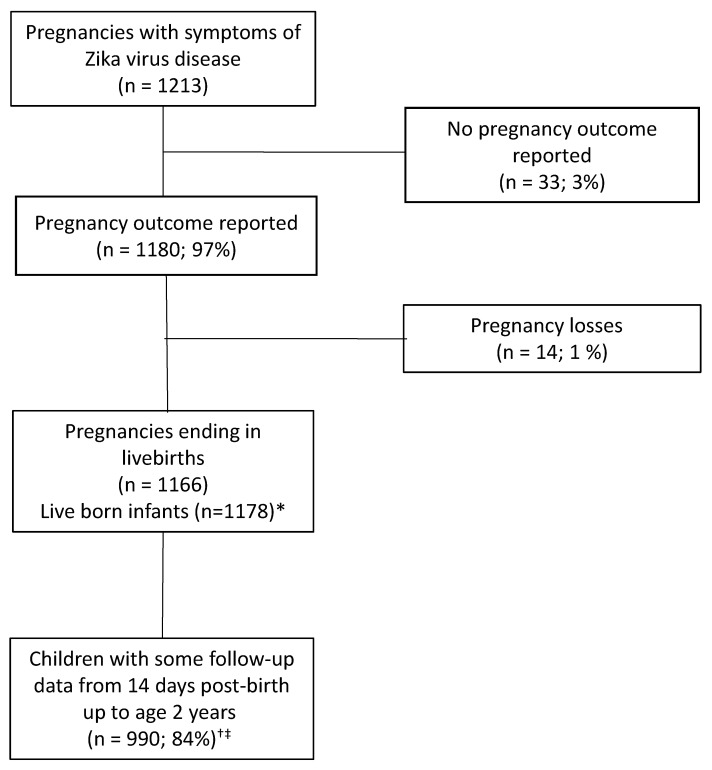
Enrollment of pregnancies and follow-up of children of two years of age, Project *Vigilancia de Embarazadas con Zika* (VEZ), Colombia, 2016–2018. * Included 12 twin pregnancies † Denominator for percentage is 1178 live born infants ‡ Follow-up data include information for 139 infants who participated in one or more of the Project VEZ brigades.

The enrollment of pregnancies and follow-up of children up to two years of age is shown in Figure 1. Of the 1213 pregnant women enrolled with symptoms of ZVD, 1180 (97.3%) were documented as having completed pregnancies with a known outcome between May 2016 and May 2017. Among the completed pregnancies, 1166 (98.8%) were deliveries of 1178 live-born infants (including 12 twin pregnancies). A total of 990 children had follow-up data available after two weeks post-birth up to two years of age.

More than half of the Project VEZ population of pregnant women were aged 20–29 years and had at least a secondary, technical, or professional education. Just over a quarter of the women were experiencing their first pregnancy. The most common ZVD symptoms reported were rashes (83.3%), fever (66.7%), and joint pain (58.6%). Just over half of the women (52.2%) reported ZVD symptom onset during the first trimester. In general, characteristics of the subcohort with laboratory evidence of a Zika virus infection were similar to the entire cohort (Table 1).

### 3.2. Pregnancy and Infant Outcomes including Zika-Associated Birth Defects

There were 50 completed pregnancies (4.2%) resulting in an infant or fetus with evidence of Zika-associated birth defects; when restricted to the 254 completed pregnancies with laboratory evidence of Zika virus infection, the proportion of fetuses/infants with Zika-associated birth defects was 8.7%. Seventeen additional infants had postnatal-onset microcephaly with no report of brain abnormalities and no evidence of microcephaly at birth. Additional clinical details for these 67 fetuses and infants with evidence of a Zika-associated birth defect or postnatal-onset microcephaly are in Table S2. (Of note, only 3 of the 19 infants with microcephaly only at birth and 2 of the 17 infants with postnatal-onset microcephaly had any neuroimaging documented in the medical record; one infant was excluded due to a neural tube defect.) Among liveborn singletons with no Zika-associated birth defects, there were 106 preterm deliveries (9.7%), 76 infants born with low birth weight (6.9%), and 50 infants born small for gestational age (4.6%). Sixteen infant deaths were reported in the cohort overall (1.4%) (Table 2).

There were no statistically significant differences by trimester of ZVD symptom onset in the proportion of pregnancies with fetuses or infants with selected eye anomalies, microcephaly only at birth, postnatal-onset microcephaly, preterm delivery, low birth weight, or small for gestational age. However, there is a pattern suggesting increased risk with earlier trimester of ZVD symptom onset for preterm delivery and low birth weight. Statistically significant differences in the proportion of selected brain anomalies with or without microcephaly were observed by trimester of ZVD symptom onset (Table 3).

### 3.3. Zika Virus Testing Results

Among the 1180 completed pregnancies, a total of 569 (48.2%) had Zika virus testing results. Pregnant women without any laboratory testing results were more likely to have secondary and technical education than those with laboratory testing results. They were also more likely to be multigravida compared to those with laboratory testing results and more likely to have delivered by Caesarean section. Finally, those without any laboratory testing results were more likely to have had periconceptional (up to 8 weeks before the estimated date of conception) ZVD symptoms compared to those with laboratory testing results (Table S3).

There were 254 pregnant women (44.6% of those with testing results) with laboratory evidence of Zika virus infection (confirmed (*n* = 220) or presumptive (*n* = 34)). As noted in the Methods, VEZ participants originally identified through national surveillance (Sivigila) had a blood sample collected as soon as possible after symptom onset and per INS protocol, testing was to be conducted within five days of symptom onset. However, for VEZ participants not identified through Sivigila, the time between symptom onset and sample collection could be much longer. The time between the date of symptom onset and date of sample collection was up to 155 days among the sample of Zika-positive pregnancies, with one or both dates missing for nearly half of the samples. There were 315 pregnant women (55.4% of those with testing results) that tested negative for Zika virus infection. Of the 611 (51.8%) participants in Project VEZ with no Zika virus testing results available, 597 (97.7%) had documentation of a sample collected during pregnancy. Of these, 184 (30.8%) had only a tissue sample collected as part of Project VEZ (i.e., umbilical cord, placenta, or fetal tissue) for which testing was not performed due to constraints in resources. The remaining 413 participants did not have a sample collected as part of Project VEZ but did have documentation in either the medical record or in Sivigila that a specimen had been taken during the pregnancy; however, there were no test results available for abstraction (Table S4).

### 3.4. Early Childhood Neurodevelopmental Outcomes

Among the 1178 children, 500 (42.4%) had documentation of any brain imaging including a cranial ultrasound, computed tomography, or magnetic resonance imaging, 605 (51.4%) had documentation of at least one ophthalmologic exam, 655 (55.6%) had documentation of at least one hearing screening, 886 (75.2%) had documentation of at least one developmental screening, and 1125 (95.8%) had at least one physical examination (data not shown).

There were 990 children in the Project VEZ cohort who had information beyond their birth hospitalization or after 14 days after birth (for children who remained in the hospital for more than 14 days after delivery) in their medical records for whom early childhood neurodevelopmental outcomes could be assessed. Of them, 126 children had documentation of any neurodevelopmental sequelae in the medical record or clinical notes from a health brigade (12.7%). Among those with Zika-associated birth defects or postnatal-onset microcephaly (*n* = 58), the proportion with any neurodevelopmental sequela documented was 44.8%; the proportion among those with neither Zika-associated birth defects nor postnatal-onset microcephaly (*n* = 932) was 10.7%. In the subgroup of infants with laboratory evidence of confirmed or presumptive Zika virus infection during the pregnancy and neither Zika-associated birth defects nor postnatal-onset microcephaly (*n* = 203), the proportion with any neurodevelopmental sequela was higher (25.1%, based on 51 infants) than among those without Zika-associated birth defects overall (10.7%). Overall, 22.4% (222/990) of children had a potential delay in achieving at least one developmental milestone. Among the 58 children with Zika-associated birth defects or postnatal-onset microcephaly, 34.5% (*n* = 20) had a potential delay in achieving at least one developmental milestone; 31.0% had a potential delay in achieving a developmental milestone in the gross motor domain, 27.6% had a potential delay in the fine motor domain, 24.1% had a potential delay in the personal and social domain, and 27.6% had a potential delay in the hearing and language domain based on the *Escala Abreviada de Desarrollo* (EAD-1) [34] at any time up to two years of age. By comparison, among children without Zika-associated birth defects, the proportion with potential delays by domain was 15–17% at any time up to age 2 years (Table 4).

## 4. Discussion 

Project VEZ was a surveillance cohort of over 1200 pregnant women with symptomatic ZVD in three cities in Colombia during the peak of the Zika virus outbreak. This cohort constituted nearly 7% of the total pregnant women diagnosed with ZVD in Colombia during the outbreak [24]. More than 97% of the pregnancies were followed until pregnancy completion and 4.2% of infants were born with brain or eye defects, including microcephaly at birth. In the subpopulation with confirmed or presumptive Zika virus infection in pregnancy, the proportion with these defects was 8.7%. This estimate is higher than what has been previously published based on passive national surveillance data from Colombia (2%) [24] but is consistent with other cohorts of pregnancies with laboratory evidence of confirmed or possible Zika virus infection [6,18,35,36]. Although these birth defects occurred among women with symptoms of ZVD, we cannot determine whether individual defects were caused by Zika virus infection or other factors. We abstracted information about possible etiology for birth defects from the medical records, and accounted for this information, where possible, in the clinical case review process, but there could have been information about alternative etiologies that was never captured during surveillance. A recent analysis of 858 cases of microcephaly or central nervous system defects reported to Colombia’s birth defects surveillance system between September 2015 and April 2017 classified 503 (59%) as potentially attributable to Zika virus infection [37]. Our findings with respect to preterm delivery and low birth weight are consistent with recently published Colombia national Zika surveillance data in which pregnancies with an earlier trimester of ZVD symptom onset appear to be at greater risk for these adverse outcomes [24]. Overall, the frequencies of preterm delivery and low birth weight were lower in the VEZ cohort than general population estimates for Colombia [38].

Although not all children received the recommended assessments, 44.8% of those with Zika-associated birth defects had at least one early childhood neurodevelopmental sequela and 34.5% had a potential developmental delay in achieving milestones in at least one EAD-1 domain. Data from the Zika Outcomes and Development in Infants and Children (ZODIAC) investigation in Brazil showed that among 19 children aged 19–24 months with Zika-associated microcephaly, 100% had a least one adverse health or developmental outcome including feeding challenges, sleeping difficulties, severe motor impairment, vision and hearing abnormalities, and seizures [12]. In a cohort of Brazilian children with microcephaly at birth related to congenital Zika virus infection, abnormalities in neurological exams were found in 97% of the children, seizures in 56.3%, and arthrogryposis in 10.8% [13]. In an analysis of data from the U.S. territories, among 87 babies with Zika associated brain and eye defects born to women with laboratory evidence of confirmed or possible Zika virus infection in pregnancy, 20 (22.9%) also had a neurodevelopmental abnormality possibly associated with congenital Zika virus infection [18]. Our findings similarly suggest that children with Zika-associated birth defects may have notable neurodevelopmental sequelae and/or potential developmental delays.

Among children without Zika-associated birth defects, there was limited evidence of adverse early childhood neurodevelopmental outcomes; nearly 22% of children screened with EAD-1 had a potential developmental delay in one or more domains (202/932) and this was somewhat higher than expected in the Colombian population [34]. However, there is inconsistency in the literature around the potential neurodevelopmental impacts of Zika virus infection in pregnancy among children without Zika-associated brain and eye defects. There is evidence of neurodevelopmental delay based on standardized assessments of cohorts of children in Brazil [22,39,40,41], Colombia [11], Nicaragua [42], Mexico [43], and French Guiana [23] but in another Brazilian cohort, there was no evidence of cognitive, language or motor delay in 18-month-old children exposed to the Zika virus during pregnancy without Zika-associated birth defects [44].

### 4.1. Limitations

The findings in this report should be evaluated in light of several limitations. First, more than half of the pregnancies in Project VEZ did not have any laboratory testing results. For 427 of these pregnancies, there were no blood or tissue samples taken during the course of the project, despite specimen collection being a component of the surveillance protocol. For the remaining 184 pregnancies, the only specimen collected was placental or umbilical cord tissue taken at delivery, for which capacity and resources were not available to complete testing. However, for nearly 50% of the Project VEZ cohort, there were laboratory testing results, either from a specimen collected as part of the enhanced surveillance protocol, or from a specimen collected as part of national surveillance. The subpopulation with laboratory results is qualitatively similar and largely representative of the full Project VEZ cohort, although some differences have been noted. The limitations of laboratory testing in general are important to note. Optimal timing of NAAT to detect Zika viral RNA in maternal serum and urine samples is seven days after symptom onset [45]. However, nearly two-thirds of the maternal serum and urine samples were taken outside of the optimal window; therefore, the NAAT results might have underestimated the true rate of confirmed Zika virus infection in the cohort. Related to this limitation, there is no “unexposed” comparison cohort within Project VEZ. Second, under-ascertainment of birth defects or neurodevelopmental sequelae was possible. The surveillance was limited to information available in the delivery or infant medical record. If infants were never seen by a clinical provider or were seen by a provider outside of the surveillance catchment area, the surveillance system would not capture that information, although Project VEZ worked closely with jurisdictional health authorities to ascertain data within the region, when possible. Documentation in the medical record of any brain imaging including a cranial ultrasound, computed tomography, or magnetic resonance imaging was present for only about 40% of the infants. In the absence of imaging, specific brain abnormalities could not be accurately identified. One of the goals of the health brigades was to capture clinical information on affected children who may not have been receiving recommended health services at the participating VEZ clinics. Despite these concerted efforts to collect clinical data, confirmation of some previous diagnoses was limited. For example, only one of four infants noted to have holoprosencephaly in the medical record had sufficient information from imaging or clinical examination to confirm the diagnosis. Because we could not be certain to have ascertained all clinical records, a lack of documentation of imaging or a clinical exam could have been a limitation of the surveillance itself and not indicative of a lack of clinical care itself. Third, although the EAD-1 is a screening instrument validated within the Colombian population, its findings cannot be compared with other internationally recognized tools. As researchers attempt to understand the longer-term neurodevelopmental effects of Zika virus infection in pregnancy using relatively small cohorts from different countries, the use of different standardized tools across countries may hinder the ability to compare across cohorts. In addition, the EAD-1 is a screener used to evaluate possible developmental delay, which would then need further clinical evaluation. The application of EAD-1 was not always standardized; children were assessed at different time points and Project VEZ, as surveillance, captured the data as noted in the record with the potential for differential assessment. In addition, because EAD-1 is a screener, it is not used to make a clinical diagnosis of developmental delay. Fourth, we have no information on asymptomatic infections; Project VEZ was surveillance based on symptomatic ZVD. The prevalence of asymptomatic Zika virus infections varies greatly [46]. However, based on other studies that have included pregnancies with both symptomatic and asymptomatic infection, the expectation is that the risk of Zika-associated birth defects would be similar regardless of symptom status [6]. A recent analysis of national Colombian surveillance data indicated that the prevalence of brain and eye defects was higher during the time of the Zika outbreak in the Americas compared to a baseline period of 2014 [24]. However, there is no baseline prevalence estimate for the adverse neurodevelopmental outcomes monitored in Project VEZ against which a comparison can be made. Finally, because of recognized error in the measurement of head circumference [47,48], as well as limitations in gestational age dating in the absence of first trimester ultrasound data, we cannot rule out the possibility of misclassification in the assessment of microcephaly.

### 4.2. Strengths

This analysis had several strengths. First, Project VEZ was a large cohort of pregnant women with symptomatic ZVD during the height of the Zika virus outbreak in Colombia. Of the over 1200 pregnancies enrolled in Project VEZ, a pregnancy outcome was ascertained for 97%, and over 75% of the infants had some clinical follow-up documented beyond the birth hospitalization. Second, because Project VEZ was built on the foundations of a strong public health surveillance infrastructure in Colombia, there was interest and engagement in Project VEZ from public health authorities at the jurisdictional and national levels. Third, because INS wanted to ensure that as many infants as possible received the recommended screenings and assessments through health brigades, additional data were captured for 139 children that would not have otherwise been available. Finally, although EAD-1 is a developmental screener rather than a diagnostic tool, it is standardized, validated, and normalized on a diverse sample of Colombian children. Our results can be compared to results from other Colombian cohorts of children in which the EAD-1 has been implemented.

## 5. Conclusions

Critical enhancements to Colombia’s existing national surveillance early in the outbreak allowed for the implementation of Project VEZ. In this large surveillance cohort of pregnant women with symptoms of ZVD during the height of the outbreak in Colombia, the proportion of fetuses/infants with any Zika-associated brain or eye defect, including microcephaly at birth, was 4.2%. This is consistent with the proportion seen in other studies to date. Among completed pregnancies of mothers with confirmed or presumptive Zika virus infection, the proportion was doubled. Despite the limitations in the completeness of Zika virus testing in this cohort, data from Project VEZ contribute to our understanding of the impact of ZVD during pregnancy. The relatively high proportion of children with a potential delay in achieving a developmental milestone in the cohort overall suggests that even among infants without Zika-associated birth defects or postnatal-onset microcephaly, exposure to ZVD in pregnancy warrants careful and comprehensive follow-up of the infant into early childhood.

## Figures and Tables

**Table 1:** Characteristics of pregnant women with symptoms of Zika virus disease and subset with laboratory evidence * of Zika virus infection, Project *Vigilancia de Embarazadas con Zika* (VEZ), Colombia, 2016–2018.

	Total (N = 1180) *n* (%)	Laboratory Evidence of Zika Virus Infection (N = 254) *n* (%)
**Maternal age at conception (years)**		
<17	106 (9.0)	28 (11.0)
17–19	208 (17.6)	41 (16.1)
20–24	374 (31.7)	76 (29.9)
25–29	249 (21.1)	55 (21.7)
30–34	149 (12.6)	34 (13.4)
≥35	78 (6.6)	17 (6.7)
Not reported	16 (1.4)	3 (1.2)
**Maternal education**		
<Secondary	120 (10.2)	30 (11.8)
Secondary	595 (50.4)	114 (44.9)
Technical	130 (11.0)	27 (10.6)
Professional	70 (5.9)	14 (5.5)
Not reported	265 (22.5)	69 (27.2)
**Gravidity**		
Primigravid	323 (27.4)	92 (36.2)
Multigravid	668 (56.6)	131 (51.6)
Not reported	189 (16.0)	31 (12.2)
**Delivery method**		
Vaginal	541 (45.9)	136 (53.5)
Cesarean section	622 (52.7)	115 (45.3)
Not reported	17 (1.4)	3 (1.2)
**Infant sex** †		
Male	575 (48.2)	124 (48.4)
Female	602 (50.5)	127 (49.6)
Not reported	15 (1.3)	5 (2.0)
**Plurality**		
Singleton	1150 (97.5)	246 (96.9)
Twins	12 (1.0)	2 (0.8)
Not reported	18 (1.5)	6 (2.4)
**Symptoms reported** ‡		
Rash	983 (83.3)	211 (83.1)
Fever	787 (66.7)	149 (58.7)
Joint pain	691 (58.6)	148 (58.3)
Headache	612 (51.9)	110 (43.3)
Conjunctivitis	267 (22.6)	54 (21.3)
Eye pain	116 (9.8)	26 (10.2)
Diarrhea	99 (8.4)	21 (8.3)
Swollen lymph nodes	22 (1.9)	5 (2.0)
Not reported	72 (6.1)	20 (7.9)
**Trimester of symptom onset**		
Periconception (up to 8 weeks before the estimated date of conception)	84 (7.1)	8 (3.2)
First trimester	616 (52.2)	124 (48.8)
Second trimester	397 (33.6)	104 (40.9)
Third trimester	63 (5.3)	13 (5.1)
Not reported	20 (1.7)	5 (2.0)

* Laboratory evidence is defined as confirmed or presumptive Zika virus infection in pregnancy. Confirmed: Zika virus nucleic acid amplification testing (NAAT) of maternal serum or urine, amniotic fluid, umbilical cord tissue, placental tissue, or fetal tissue was positive, regardless of Zika virus immunoglobulin M (IgM) test results; Presumptive: Zika virus NAAT of maternal serum or urine, amniotic fluid, umbilical cord tissue, placental tissue, or fetal tissue was negative or not performed, but maternal or fetal Zika virus IgM test results were positive and dengue virus IgM test results were negative. Of note, the interval between symptom onset and NAAT was often prolonged. † Overall denominator (*n* = 1192) includes 1178 live-born infants and 14 pregnancy losses. Denominator for subgroup with laboratory evidence (*n* = 256) includes 252 live-born infants and 4 pregnancy losses. ‡ Not mutually exclusive; participants could report more than one symptom.

**Table 2:** Pregnancy and infant outcomes among pregnant women with symptoms of Zika virus disease and subset with laboratory evidence * of Zika virus infection, Project *Vigilancia de Embarazadas con Zika* (VEZ), Colombia, 2016–2018.

	Total (N = 1180) *n* (%)	Laboratory Evidence of Zika Virus Infection (N = 254) *n* (%)
**Pregnancy outcome**		
Live birth	1166/1180 (98.8)	250/254 (98.4)
Pregnancy loss <20 weeks’ gestation	6/1180 (0.5)	1/254 (0.4)
Pregnancy loss ≥20 weeks’ gestation	8/1180 (0.7)	3/254 (1.2)
**Zika-associated birth defects**		
Total with any Zika-associated birth defect	50/1180 (4.2)	22/254 (8.7)
Selected brain abnormalities with or without microcephaly	29/1180 (2.5)	14/254 (5.5)
Selected eye anomalies	14/1180 (1.2)	6/254 (2.4)
Microcephaly only at birth	19/1180 (1.6)	7/254 (2.8)
**Postnatal-onset microcephaly**	17/1180 (1.4)	4/254 (1.6)
**Other adverse outcomes** †		
Preterm delivery ‡	106/1099 (9.7)	21/226 (9.3)
Low birth weight ‡	76/1099 (6.9)	19/226 (8.4)
Small for gestational age ‡	50/1099 (4.6)	8/226 (3.5)
Death in the first year of life (among live-born infants)	16/1178 (1.4)	9/252 (3.6)

* Laboratory evidence is defined as confirmed or presumptive Zika virus infection in pregnancy. Confirmed: Zika virus nucleic acid amplification testing (NAAT) of maternal serum or urine, amniotic fluid, umbilical cord tissue, placental tissue, or fetal tissue was positive, regardless of Zika virus immunoglobulin M (IgM) test results; Presumptive: Zika virus NAAT of maternal serum or urine, amniotic fluid, umbilical cord tissue, placental tissue, or fetal tissue was negative or not performed, but maternal or fetal Zika virus IgM test results were positive and dengue virus IgM test results were negative. Of note, the interval between symptom onset and NAAT was often prolonged. † Among singleton live births without Zika-associated birth defects; ‡ Preterm birth defined as delivery <37 completed weeks’ gestation. Low birth weight defined as birth weight <2500 g. Small for gestational age is defined as birth weight <10th percentile for gestational age and sex using INTERGROWTH-21st standard.

**Table 3:** Pregnancy and infant outcomes among pregnant women with symptoms of Zika virus disease, by trimester of symptom onset, Project *Vigilancia de Embarazadas con Zika* (VEZ), Colombia, 2016–2018.

	Total (N = 1160) *	Periconception (N = 84)	1st Trimester (N = 616)	2nd Trimester (N = 397)	3rd Trimester (N = 63)
	*n* (%)	*n* (%)	*n* (%)	*n* (%)	*n* (%)
**Pregnancy outcome**					
Live birth	1149/1160 (99.1)	81/84 (96.4)	610/616 (99.0)	395/397 (99.5)	63/63 (100.0)
Pregnancy loss <20 weeks’ gestation	4/1160 (0.3)	1/84 (1.2)	3/616 (0.5)	0/397 (0)	0/63 (0)
Pregnancy loss ≥20 weeks’ gestation	7/1160 (0.6)	2/84 (2.4)	3/616 (0.5)	2/397 (0.5)	0/63 (0)
**Zika-associated birth defects**					
Total with any Zika-associated birth defect	49/1160 (4.2)	4/84 (4.8)	29/616 (4.7)	13/397 (3.3)	3/63 (4.8)
Selected brain abnormalities with or without microcephaly	28/1160 (2.4)	2/84 (2.4)	19/616 (3.1)†	4/397 (1.0)†	3/63 (4.8)
Selected eye anomalies	14/1160 (1.2)	0/84 (0)	11/616 (1.8)	2/397 (0.5)	1/63 (1.6)
Microcephaly only at birth	19/1160 (1.6)	2/84 (2.4)	9/616 (1.5)	8/397 (2.0)	0/63 (0)
**Postnatal-onset microcephaly**	17/1160 (1.5)	3/84 (3.6)	7/616 (1.1)	6/397 (1.5)	1/63 (1.6)
**Other adverse outcomes ‡**					
Preterm delivery §	105/1082 (9.7)	10/75 (13.3)	65/573 (11.3)	26/375 (6.9)	4/59 (6.8)
Low birth weight §	75/1082 (6.9)	10/75 (13.3)	41/573 (7.2)	21/375 (5.6)	3/59 (5.1)
Small for gestational age §	50/1082 (4.6)	3/75 (4.0)	23/573 (4.0)	20/375 (5.3)	4/59 (6.8)
Death in the first year of life (among live births)	14/1149 (1.2)	0/81 (0)	10/614 (1.6)	3/395 (0.8)	1/63 (1.6)

* 20 pregnancies were missing trimester of symptom onset. † Chi-squared test comparing first and second trimester of ZVD symptom onset was statistically significant (*p* = 0.03). All other comparisons by trimester of ZVD symptom onset were not statistically significant. ‡ Among singleton live births without Zika-associated birth defects; § Preterm birth defined as delivery <37 completed weeks’ gestation. Low birth weight defined as birth weight <2500 g. Small for gestational age defined as birth weight <10th percentile for gestational age and sex using INTERGROWTH-21st standard.

**Table 4:** Early childhood neurodevelopmental outcomes * among children with follow-up data born to pregnant women with symptoms of Zika virus disease and subset with laboratory evidence † of Zika virus infection, Project *Vigilancia de Embarazadas con Zika* (VEZ), Colombia, 2016–2018.

	Total (N = 990)		Laboratory Evidence of Zika Virus Infection (N = 223)	
Neurodevelopmental Sequelae	Zika-Associated Birth Defects ‡ or Postnatal onset Microcephaly (N = 58) *n* (%)	No Zika-Associated Birth Defects (N = 932) *n* (%)	Zika-Associated Birth Defects or Postnatal onset Microcephaly (N = 20) n (%)	No Zika-Associated Birth Defects (N = 203) *n* (%)
Seizures	12 (20.7)	5 (0.5)	4 (20.0)	2 (1.0)
Tone abnormality	19 (32.8)	71 (7.6)	8 (40.0)	44 (21.7)
Movement abnormality	13 (22.4)	6 (0.6)	4 (20.0)	2 (1.0)
Swallowing abnormality	11 (19.0)	7 (0.8)	3 (15.0)	4 (2.0)
Arthrogryposis	1 (1.7)	0 (0)	1 (5.0)	0 (0)
Visual impairment	12 (20.7)	12 (1.3)	6 (30.0)	3 (1.5)
Hearing abnormality	3 (5.2)	22 (2.4)	2 (10.0)	3 (1.5)
**Any neurodevelopmental sequela**	**26 (44.8)**	**100 (10.7)**	**11 (55.0)**	**51 (25.1)**
***Escala Abreviada de Desarrollo*** **(EAD-1) § alert scores**				
Gross motor domain	18 (31.0)	159 (17.1)	4 (20.0)	21 (10.3)
Fine motor domain	16 (27.6)	142 (15.2)	4 (20.0)	19 (9.4)
Personal and social domain	14 (24.1)	152 (16.3)	4 (20.0)	17 (8.4)
Hearing and language domain	16 (27.6)	151 (16.2)	4 (20.0)	16 (7.9)
**Any EAD-1 alert**	**20 (34.5)**	**202 (21.7)**	**4 (20.0)**	**30 (14.8)**

* Early childhood neurodevelopmental outcomes were defined as neurodevelopmental sequelae (seizures, tone abnormality, movement abnormality, swallowing abnormality, arthrogryposis, visual impairment, or hearing abnormality), or evidence of potential delay in achieving a developmental milestone based on an alert score on the *Escala Abreviada de Desarrollo* (EAD-1). † Laboratory evidence is defined as confirmed or presumptive Zika virus infection in pregnancy. Confirmed: Zika virus nucleic acid amplification testing (NAAT) of maternal serum or urine, amniotic fluid, umbilical cord serum or cord tissue, placental tissue, or fetal tissue was positive, regardless of Zika virus immunoglobulin M (IgM) test results; Presumptive: Zika virus NAAT of maternal serum or urine, amniotic fluid, umbilical cord serum or cord tissue, placental tissue, or fetal tissue was negative or not performed, but maternal or fetal Zika virus IgM test results were positive and dengue virus IgM test results were negative. Of note, the interval between symptom onset and NAAT was often prolonged. ‡ Zika-associated birth defects are defined as brain abnormalities with or without microcephaly and structural eye abnormalities [31] § *Escala Abreviada de Desarrollo* (EAD-1) is a standardized, validated developmental screening tool normed on diverse sample of children in Colombia. An alert was based on cut-points defined in the EAD-1 manual. There were 15 children with Zika-associated birth defects and 284 children without Zika-associated birth defects with either no data on EAD-1 or data from a different developmental assessment tool, the results from which could not be combined with those of the EAD-1. These children are included in the denominator but only data from the EAD-1 were included in the numerator to reflect possible developmental delay.

## Data Availability

These data are collected under relevant provisions of the Public Health Service Act and are protected at CDC by an Assurance of Confidentiality (Section 308(d) of the Public Health Service Act, 42 U.S.C. §242 m(d)) (https://www.cdc.gov/od/science/integrity/confidentiality/, accessed on 26 June 2021), which prohibits use or disclosure of any identifiable or potentially identifiable information collected under the Assurance for purposes other than those set out in the Assurance. Requests for access will be considered on a case-by-case basis, and inquiries should be directed to mmercado@ins.gov.co.

## Supplementary Materials

The following are available online at https://www.mdpi.com/article/10.3390/tropicalmed6040183/s1, Table S1: Algorithm for classification of Zika virus infection based on Zika virus nucleic acid amplification test (NAAT) results and Zika virus and dengue virus immunoglobulin M (IgM) test results, Project *Vigilancia de Embarazadas con Zika* (VEZ), Colombia, 2016–2018, Table S2: Clinical details of 50 fetuses and infants with Zika-associated birth defects and 17 infants with postnatal-onset microcephaly, Project *Vigilancia de Embarazadas con Zika* (VEZ), Colombia, 2016–2018, Table S3: Characteristics among pregnant women with complete pregnancy outcome data, comparing those with any Zika virus laboratory testing results, and those with no Zika virus laboratory testing results, Project *Vigilancia de Embarazadas con Zika* (VEZ), Colombia, 2016–2018, Table S4: Zika virus laboratory testing results, and documentation of samples among those with no Zika virus laboratory testing results, Project *Vigilancia de Embarazadas con Zika* (VEZ), Colombia, 2016–2018.

## Disclaimer

The findings and conclusions in this report are those of the authors and do not necessarily represent the official position of the Centers for Disease Control and Prevention or the United States Agency for International Development. Disclosure of prior presentation of study data as an abstract or poster: The data from this study have been presented previously as a platform presentation at the Infectious Diseases Society of America 2019 Annual Meeting (IDWeek) and poster and a platform presentation at the American Society of Tropical Medicine and Hygiene 2019 Annual Meeting.
