# Supplementary material for: Pregnancy, Birth, Infant, and Early Childhood Neurodevelopmental Outcomes among a Cohort of Women with Symptoms of Zika Virus Disease during Pregnancy in Three Surveillance Sites, Project Vigilancia de Embarazadas con Zika (VEZ), Colombia, 2016–2018
Source: Trop Med Infect Dis. 2021 Oct 12;6(4):183. doi: 10.3390/tropicalmed6040183 (PMC8544689; doi:10.3390/tropicalmed6040183)
Supplement: Supplementary file 1 [file tropicalmed-06-00183-s001.zip › tropicalmed-1369424-supplementary.pdf]

Tropical Medicine and
Infectious Disease

MDPI

Supplementary material of Pregnancy, Birth, Infant, and Early Childhood Neurodevelopmental Outcomes among a Cohort of Women with Symptoms of Zika Virus Disease during Pregnancy in Three Surveillance Sites, Project *Vigilancia de Embarazadas con Zika* (VEZ), Colombia, 2016–2018

Table S1. Algorithm for classification of Zika virus infection based on Zika virus nucleic acid amplification test (NAAT) results and Zika virus and dengue virus immunoglobulin M (IgM) test results, Project *Vigilancia de Embarazadas con Zika* (VEZ), Colombia, 2016 – 2018.

1. If Zika virus NAAT* of maternal serum or urine, amniotic fluid, umbilical cord tissue, placental tissue, or fetal tissue was positive, regardless of Zika virus IgM test results, then the pregnancy was considered to have **confirmed** Zika virus infection.
2. If Zika virus NAAT* of maternal serum or urine, amniotic fluid, umbilical cord tissue, placental tissue, or fetal tissue was negative or not performed, but maternal or fetal Zika virus IgM test results were positive and dengue virus IgM† test results were negative, then the pregnancy was considered to have **presumptive** Zika virus infection.
3. If all Zika virus NAAT* of maternal serum or urine, amniotic fluid, umbilical cord tissue, placental tissue, or fetal tissue

* The interval between symptom onset and NAAT was often prolonged.

† Dengue virus IgM was only performed on Zika virus IgM positive samples.

Table S2. Clinical details of 50 fetuses and infants with Zika-associated birth defects including microcephaly only at birth, and 17 infants with postnatal-onset microcephaly, Project *Vigilancia de Embarazadas con Zika* (VEZ), Colombia, 2016 – 2018.

**Case**	**Trimester of maternal Zika virus (ZIKV) symptoms**<br><br>**Maternal ZIKV testing results**	**Gestational age at delivery in weeks**<br><br>**Infant sex**<br><br>**Vital status at delivery**	**Growth parameters at birth*:**<br><br>**HC, cm (percentile)**<br><br>**Weight, g (percentile)**<br><br>**Length, cm (percentile)**	**Main neuroimaging type and findings**	**Neurodevelopmental sequelae**	***Escala Abreviada del Desarrollo* Alerts (age range and domain of alert noted)**	**Other findings**
**1**	Second trimester<br><br>Presumptive ZIKV infection	37 weeks<br><br>Male<br><br>Live birth	HC: 33 (49.4)<br><br>Weight: 2800 (40.8)<br><br>Length: 49 (74.7)	Not available	**Eye/vision:** normal<br><br>**Hearing:** Not available<br><br>**Neurologic:** Microcephaly in infant follow-up	No alerts in developmental screening	No additional findings
**2**	No ZIKV testing available	38 weeks<br><br>Female<br><br>Live birth	HC: 34 (80)<br><br>Weight: 3640 (94.3)<br><br>Length: 51 (96.6)	Not available	**Eye/vision:** Not available<br><br>**Hearing:** Not available<br><br>**Neurologic:** Microcephaly in infant follow-up	Not available	No additional findings
**3**	Third trimester<br><br>Presumptive ZIKV infection	36 weeks<br><br>Male<br><br>Live birth	HC: 28 (0.04)<br><br>Weight: 2420 (24.7)<br><br>Length: 48 (71.3)	**Prenatal US:** Ventriculomegaly, intracranial calcifications, microcephaly, abnormal cortical gyral patterns<br><br>**Cranial US:** Thin cortical mantle, ventriculomegaly, intraventricular calcifications, absent corpus callosum<br><br>**Cranial CT**: Microcephaly, calcifications in frontal and parietal lobes and cerebellum thinning of the cerebral cortex in frontal lobe, absent corpus	**Eye/vision:** Normal<br><br>**Hearing:** Not available<br><br>**Neurologic:** Microcephaly at birth and infant follow-up, seizures, increased body tone	Gross motor alert (6-11 & 12-17 months)<br><br>Fine motor alert (6-11 & 12-17 months)<br><br>Hearing and language alert (6-11 & 12-17 months)<br><br>Personal and social alert (6-11 & 12-17 months)	Cryptorchidism<br><br>Prominent occipital bone, craniofacial disproportion

				callosum, absent cerebral sulci, probable lissencephaly, dilated lateral and posterior ventricular horns (ventriculomegaly)			
**4**	First trimester<br><br>No laboratory evidence of ZIKV infection	41 weeks<br>Female<br>Live birth	HC: 31 (0.29)<br><br>Weight: 3520 (64.3)<br><br>Length: 50 (56.5)	**Prenatal US:** Intracranial calcifications<br><br>**Brain MRI:** Microcephaly Cortical thinning, gliosis in white matter, periventricular leukomalacia<br><br>**Cranial US**: Normal<br><br>**Cranial CT**: Multiple intracranial calcifications in frontal and parietal lobes	**Eye/vision**: Possible visual impairment (bilateral strabismus)<br><br>**Hearing**: Normal exam<br><br>**Neurologic:** Microcephaly at birth and follow up, seizures, swallowing difficulty	Gross motor alert (0-5, 6-11, 12-17, & 18-24 months)<br><br>Fine motor alert (6-11, 12-17, & 18-24 months)<br><br>Hearing and language alert (6-11, 12-17, & 18-24 months)<br><br>Personal and social alert (12-17 & 18-24 months)	Narrow forehead, extra skin on head, craniofacial disproportion, absent frenulum, prominent frontal bone
**5**	First trimester<br><br>Confirmed ZIKV infection	39 weeks<br>Male<br>Live birth	HC: 30 (0.09)<br><br>Weight: 2345 (1.4)<br><br>Length: 48 (21.8)	**Cranial US:** Normal	**Eye/vision:** Not available<br><br>**Hearing:** Not available<br><br>**Neurologic:** Microcephaly	Not available	SGA
**6**	Second trimester<br><br>Confirmed ZIKV infection	40 weeks<br><br>Female<br><br>Live birth	HC: 32 (5.12)<br><br>Weight: 2290 (0.62)<br><br>Length: 41 (0.00)	**Cranial US:** Normal	**Eye/vision:** Normal<br><br>**Hearing:** Normal<br><br>**Neurologic:** Microcephaly in infant follow-up, tone abnormalities. Microcephaly	Gross motor alert (18-24 months)	SGA
**7**	Second trimester<br><br>No ZIKV testing results	38 weeks<br><br>Male<br><br>Live birth	HC: no data<br><br>Weight: 2800 (24.3)<br><br>Length: 47 (17.6)	Not available	**Eye/vision:** not available<br><br>**Hearing:** normal<br><br>**Neurologic:** microcephaly in infant follow-up	Not available	No additional findings
**8**	First trimester<br><br>Presumptive ZIKV infection	40 weeks<br><br>Male<br><br>Live birth	HC: 35 (72.6)<br><br>Weight: 2940 (12.8)	**Cranial US:** Ventriculomegaly	**Eye/vision:** Not available<br><br>**Hearing:** Not available	Not available	Not available

			Length: 48 (11.9)		**Neurologic:** Not available		
**9**	First trimester<br><br>Presumptive ZIKV infection	36 weeks<br><br>Male<br><br>Live birth	HC: 31 (10.97)<br><br>Weight: 2315 (17.1)<br><br>Length: 46 (29.7)	**Prenatal US**: IUGR, microcephaly, colpocephaly, bilateral ventriculomegaly, Dandy Walker anomaly, Blake pouch cyst, bilateral talipes equinovarus	**Eye/vision:** Not available<br><br>**Hearing:** Not available<br><br>**Neurologic**: Not available	Not available	Short neck, pectus excavatum, hip dystocia, overlapping toes, talipes equinovarus.<br>Infant died at 12 days of age
**10**	First Trimester<br><br>No ZIKV testing results	40 weeks<br>Female<br>Live birth	HC: 28(0.00)<br><br>Weight: 2900 (16.9)<br><br>Length: 49 (44.1)	**Prenatal US**: Microcephaly, ventriculomegaly, periventricular calcifications, abnormal corpus callosum<br><br>**Cranial CT**: Ventriculomegaly, possible dysgenesis of corpus callosum<br><br>**Brain MRI**: Thin cortical mantle, ex vacuo enlargement of lateral ventricles	**Eye/vision:** Abnormal retinal pigment<br><br>**Hearing**: Not available<br><br>**Neurologic:** Microcephaly at birth and follow-up, swallowing difficulty, seizures, increased body tone, movement abnormalities	Gross motor alert (6-11, 12-17, & 18-24 months)<br><br>Fine motor alert (6-11, 12-17, & 18-24 months)<br><br>Hearing and language alert (6-11, 12-17, & 18-24 months)<br><br>Personal and social alert (6-11, 12-17, & 18-24 months)	Asymmetric cranium
**11**	Periconception<br><br>No ZIKV testing results	34 weeks<br><br>Female<br><br>Live birth	HC: 33(92.4)<br><br>Weight: not available<br><br>Length: not available	**Cranial CT**: Dysgenesis of the corpus callosum, and periventricular calcifications. Ventriculomegaly	**Eye/vision:** Normal<br><br>**Hearing**: Not available<br><br>**Neurologic:** Not available	Not available	Infant positive and amniotic fluid +
**12**	First trimester<br><br>Confirmed ZIKV infection	28 weeks<br>Male<br>Live birth	HC: Not available<br><br>Weight: 1270 (80.9)<br><br>Length: 38 (60.7)	**Prenatal US:** Microcephaly, closure of anterior and posterior fontanels (craniosynostosis), hypoechogenicity and reduction of the frontal lobe of the brain (hypoplasia), microcalcifications	**Eye/vision:** Retinal calcifications<br><br>**Hearing:** Not available<br><br>**Neurologic:** Not available	Not available	Hypogonadism, dysmorphic features, craniosynostosis<br><br>Infant died on date of birth<br><br>Infant brain tissue pathology evaluation: thinning of the cerebral cortex with apoptosis and microcalcifications

				**Cranial US:** Cerebral atrophy, intracranial calcifications, hypoplasia of frontal lobe, vasculitis			
**13**	First trimester<br><br>No ZIKV testing results	36 weeks<br>Male<br>Live birth	HC: 29 (0.36)<br><br>Weight: 2400 (23.2)<br><br>Length: 46 (29.7)	**Cranial US**: Intracranial calcifications (periventricular and subcortical), dysgenesis of corpus callosum, abnormal gyri, ventriculomegaly<br><br>**Cranial CT**: Increased ventricular size especially of posterior horns. Cortico-subcortical and basal ganglia calcifications, absent sulci, thin cerebral cortex.<br><br>**Brain MRI**: Simplified gyral pattern, ventriculomegaly, agenesis of corpus callosum	**Eye/vision:** Microcornea, chorioretinal lesions extramacular scar in left eye. Nystagmus and strabismus<br><br>**Hearing:** Normal evaluation<br><br>**Neurologic:** Microcephaly at birth and follow-up, seizures, tone and movement abnormalities, swallowing difficulties	Gross motor alert (6-11, 12-17, & 18-24 months)<br><br>Fine motor alert (6-11, 12-17, & 18-24 months)<br><br>Hearing and language alert (6-11, 12-17, & 18-24 months)<br><br>Personal and social alert (6-11, 12-17, & 18-24 months)	Narrow forehead, overlapped sutures, extra skin folds on neck, high arched palate, micrognathia,
**14**	First trimester<br><br>No laboratory evidence of ZIKV infection	35 weeks<br>Male<br>Live birth	HC: 30 (5.95)<br><br>Weight: 2465 (48.9)<br><br>Length: 46 (49.5)	**Prenatal US:** Ventriculomegaly, mega cisterna magna, probable dysgenesis of the corpus callosum<br><br>**Brain MRI:** Thin cortical mantle, thin corpus callosum, ventriculomegaly,	**Eye/vision:** Abnormal retinal pigment, optic nerve hypoplasia<br><br>**Hearing**: Normal<br><br>**Neurologic:** Microcephaly in infant follow-up, seizures, tone abnormality, movement abnormality	Gross motor alert (6-11, 12-17, & 18-24 months)<br><br>Fine motor alert (6-11, 12-17, & 18-24 months)<br><br>Hearing and language alert (6-11, 12-17, & 18-24 months)<br><br>Personal and social alert (6-11, 12-17, & 18-24 months)	Abnormal appearance of head
**15**	First trimester	28 weeks<br>Male<br>Live birth	HC: 23 (3.19)	**Prenatal US**: Ventriculomegaly, talipes equinovarus	**Eye/vision:** Not available	Not available	Club foot, esophageal atresia, abnormal facies, pulmonary malformations

	No laboratory evidence of ZIKV infection		Weight: 910 (19.7)<br><br>Length: 33 (4.93)		**Hearing**: Not available<br><br>**Neurologic:** Not available		Infant death
**16**	First trimester<br><br>No laboratory available	39 weeks<br><br>Female<br><br>Live birth	HC: Not available<br><br>Weight: Not available<br><br>Length: Not available	Not available	**Eye/vision:** Not available<br><br>**Hearing**: Not available<br><br>**Neurologic:** microcephaly in infant follow-up	No alerts in developmental screening	No additional findings
**17**	First trimester<br><br>No laboratory evidence of ZIKV infection	38 weeks<br>Male<br>Live birth	HC: 30 (0.28)<br><br>Weight: 2800 (24.2)<br>Length: 51 (91.5)	**Brain MRI:** Hypoplasia of corpus callosum, cerebral atrophy, ventriculomegaly, lissencephaly<br><br>**Cranial CT**: Decrease size of cranial vault, dilatation of the supra and infratentorial ventricular systems, decrease of cortical trails in relation to atrophic changes associated with cortical immaturity, discrete decrease in the differentiation of gray and white matter, mega-cisterna magna-like variant, brain atrophy, areas of lissencephaly and pachygyria, hypoplastic corpus callosum	**Eye/vision:** Bilateral pale and hypoplastic optic nerves, bilateral abnormal retinal pigment dispersion, vision impairment<br><br>**Hearing**: Normal evaluation<br><br>**Neurologic:** Microcephaly at birth and follow-up, seizures, movement abnormality, tone abnormality, swallowing difficulties	Gross motor alert (6-11, 12-17, & 18-24 months)<br><br>Fine motor alert (6-11, 12-17, & 18-24 months)<br><br>Hearing and language alert (6-11, 12-17, & 18-24 months)<br><br>Personal and social alert (6-11, 12-17, & 18-24 months)	Non-palpable fontanelles, overlapping sutures, low set ear (left), narrow forehead, high arched palate, micrognathia, mildly depressed nasal bridge
**18**	Periconception<br><br>Presumptive ZIKV infection	39 weeks<br>Female<br>Live birth	HC: 28 (0.00)<br><br>Weight: 2400 (2.62)	**Cranial US:** Ventriculomegaly<br><br>**Cranial CT:** Microcephaly,	**Eye/vision:** Not available.<br><br>**Hearing:** Not available<br><br>**Neurologic:** Microcephaly	Not available	SGA

			Length: 44 (0.42)	cerebral atrophy, ventriculomegaly, intracranial calcifications, (subcortical), absent cavum septum pellucidum, cortical atrophy			
**19**	First trimester<br><br>Presumptive ZIKV infection	37 weeks<br>Male<br>Live birth	HC: 32 (20.03)<br><br>Weight: 2810 (41.8)<br><br>Length: 47 (32.0)	**Cranial US:** Parenchymal calcifications, small cerebellum<br><br>**Cranial CT:** Intracranial calcifications<br><br>**Brain MRI:** Bilateral atrophy of the frontal lobes, right frontal polymicrogyria	**Eye/vision:** Normal<br><br>**Hearing:** Normal<br><br>**Neurologic:** Microcephaly in infant follow-up swallowing abnormality, tone abnormality, seizures movement	Gross motor alert (0-5, 6-11, 12-17, & 18-24 months)<br><br>Fine motor alert (0-5, 6-11, 12-17, & 18-24 months)<br><br>Hearing and language alert (0-5, 6-11, 12-17, & 18-24 months)<br><br>Personal and social alert (0-5, 6-11, 12-17, & 18-24 months)	No additional findings
**20**	First trimester<br><br>Presumptive ZIKV infection	33 weeks<br>Female<br>Live birth	HC: 26 (0.10)<br><br>Weight: 1105 (1.66)<br><br>Length: 38 (0.37)	**Cranial US:** Intracranial calcifications bilateral, mild ventricular dilatation	**Eye/vision:** Retinopathy, bilateral eye proptosis, chorioretinitis in macula,<br><br>**Hearing:** Not available<br><br>**Neurologic:** Microcephaly, arthrogryposis, clench hands and right upper limb hemiparesis, seizures	Not available	Dysmorphic features, missing a phalange in 5th finger left hand, bilateral camptodactyly, simian crease, low set ears.<br><br>Infant died at 4 months of age<br><br>SGA
**21**	Second trimester<br><br>Confirmed ZIKV infection	22 weeks<br>Infant sex not available<br>Fetal loss	HC: Not available<br><br>Weight: Not available<br>Length: Not available	**Prenatal US:** Ventriculomegaly, corpus callosum abnormality, cerebral atrophy, Alobar holoprosencephaly†	**Eye/vision:** Not available<br><br>**Hearing:** Not available<br><br>**Neurologic:** Not available	Not available	Not available
**22**	Third trimester<br><br>No ZIKV testing results	37 weeks<br><br>Female<br><br>Live birth	HC: not available<br><br>Weight: 1900 (0.81)<br><br>Length: 44 (2.98)	Not available	**Eye/vision:** Not available<br><br>**Hearing:** Not available<br><br>**Neurologic**: Postnatal microcephaly	Gross motor alert (6-11 & 12-17 months)<br><br>Fine motor alert (12-17 months)	SGA
**23**	First trimester	37 weeks<br><br>Male	HC: 38 (99.9)	**Brain MRI**: Intracranial calcifications,	**Eye/vision:** Not available	Not available	Not available

	No ZIKV testing results	Live birth	Weight: 3620 (95.0)<br><br>Length: 49 (74.7)	cerebral cortical atrophy, prominent subarachnoid space	**Hearing:** Not available<br><br>**Neurologic**: Seizures		
**24**	Periconception<br><br>No ZIKV testing results	39 weeks<br><br>Male<br><br>Live birth	HC: 34 (53.4)<br><br>Weight: 3000 (27.2)<br><br>Length: 49 (43.0)	Not available	**Eye/vision:** Not available<br><br>**Hearing:** Not available<br><br>Neurologic: microcephaly in infant follow-up	No alerts in developmental screening	Not available
**25**	Second trimester<br><br>No ZIKV testing results	39 weeks<br><br>Female<br><br>Live birth	HC: Not available<br><br>Weight: 2800 (18.9)<br><br>Length: 49 (58.7)	Not available	**Eye/vision:** Not available<br><br>**Hearing:** Not available<br><br>**Neurologic:** microcephaly in infant follow-up	No alerts in developmental screening	Not available
**26**	First trimester<br><br>No ZIKV testing results	39 weeks<br>Female<br>Live birth	HC: 31 (1.62)<br><br>Weight: 2100 (0.40)<br><br>Length: 51 (93.0)	Not available	**Eye/vision:** Not available<br><br>**Hearing:** Not available<br><br>**Neurologic:** Microcephaly	No alerts in developmental screening	SGA
**27**	Second trimester<br><br>No ZIKV testing results	40 weeks<br>Male<br>Live birth	HC: not available<br><br>Weight: 2500 (1.44)<br><br>Length: 49 (28.2)	Not available	**Eye/vision:** Not available<br><br>**Hearing:** Not available<br><br>**Neurologic:** Microcephaly in infant follow-up	Gross motor alert (6-11 & 18-24 months)<br><br>Fine motor alert (18-24 months)<br><br>Personal and social alert (18-24 months)<br><br>Hearing and language alert (18-24 months)	SGA
**28**	Second trimester<br><br>No ZIKV testing results	37 weeks<br><br>Female<br><br>Live birth	HC: 37 (99.9)<br><br>Weight: 3020 (71.3)<br><br>Length: 47 (43.1)	**Prenatal US:** hydrocephaly	**Eye/vision:** Not available<br><br>**Hearing:** Not available<br><br>**Neurologic:** Not available	Not available	No additional findings
**29**	Third trimester<br><br>No ZIKV testing results	38 weeks<br><br>Female<br><br>Live birth	HC: not available<br><br>Weight: 3475 (88.8)<br><br>Length: 46 (11.3)	Not available	**Eye/vision:** Not available<br><br>**Hearing:** Not available<br><br>**Neurologic:** Microcephaly in infant follow-up	No alerts in developmental screening	No additional findings
**30**	First trimester<br><br>No ZIKV testing results	37 weeks<br>Male<br>Live birth	HC: 30 (0.83)	Not available	**Eye/vision:** Not available<br><br>**Hearing:** Not available	No alerts in developmental screening	No additional findings

			Weight: 3085 (67.8)<br><br>Length: 49 (74.7)		**Neurologic:** Microcephaly		
**31**	Periconception<br><br>No ZIKV testing results	40 weeks Female Live birth	HC: 31 (0.68)<br><br>Weight: 2890 (16.2)<br><br>Length: 45 (0.68)	**Prenatal US:** normal	**Eye/vision:** Not available<br><br>**Hearing:** Not available<br><br>**Neurologic:** Microcephaly at birth and in infant follow-up	Gross motor alert (6-11 months)	No additional findings
**32**	Second trimester<br><br>Confirmed ZIKV infection	38 weeks Male Live birth	HC: 31 (2.06)<br><br>Weight: 2597 (11.2)<br><br>Length: 45 (2.32)	**Prenatal US:** Intracardiac echogenic spot, otherwise normal<br><br>**Cranial US**: Normal	**Eye/vision:** Not available<br><br>**Hearing**: Not available<br><br>**Neurologic:** Microcephaly at birth	No alerts in developmental screening	No additional findings
**33**	First trimester<br><br>No laboratory evidence for ZIKV infection	37 weeks Male Live birth	HC: 29 (0.10)<br><br>Weight: 3240 (79.4)<br><br>Length: 47 (32.0)	**Prenatal Ultrasound**: Hypoplasia of nasal bone<br><br>**Cranial US**: Microcephaly, subcortical calcifications, cerebral and cerebellar abnormality, hydrocephalus<br><br>**Cranial CT**: Cerebral, basal ganglia, brain stem and cerebellar atrophy, subcortical calcifications, communicating hydrocephaly, cerebral and cerebellar hypoplasia, and cerebral hypogyration<br><br>**Brain MRI**: cerebellar atrophy, non-communicating hydrocephalus, thinning of both hemispheres of the brain, smooth appearance of the cortical grooves,	**Eye/vision:** Bilateral red spot in retina, bilateral chorioretinitis, vision impairment<br><br>**Hearing**: Not available<br><br>**Neurologic:** Microcephaly at birth and in infant follow-up, seizures, tone, movement and movement swallowing abnormalities	Personal social alert (18-24 months)	Dysmorphic facial features, low set ears

				thalamic and brain stem hypoplasia			
**34**	Second trimester<br><br>No laboratory evidence of ZIKV infection	37 weeks<br>Female<br>Live birth	HC: not available<br><br>Weight: 2094 (2.73)<br><br>Length: 44 (2.98)	**Prenatal US**: Bilateral ventriculomegaly, agenesis of corpus callosum, cortical calcifications<br><br>**Brain MRI**: dysgenesis of corpus callosum, posterior horn dilatation, absent septum pellucidum	**Eye/vision:** Not available<br><br>**Hearing**: Not available<br><br>**Neurologic:** Microcephaly, swallowing difficulty	Gross motor alert (12-17 months)<br><br>Fine motor alert (12-17 months)<br><br>Hearing and language alert (12-17 months)	SGA
**35**	First trimester<br><br>No ZIKV testing results	20 weeks<br>Fetal sex not available<br><br>Fetal loss	HC: not available<br><br>Weight: not available<br><br>Length: not available	**Prenatal US:** Ventriculomegaly, hypoplasia of vermis, absent midline brain structures. Microcephaly. Arthrogryposis	**Eye/vision:** Not available<br><br>**Hearing**: Not available<br><br>**Neurologic:** Not available	Not available	Not available
**36**	Second Trimester<br><br>No ZIKV testing results	39 weeks<br>Male<br>Live birth	HC: 31 (0.78)<br><br>Weight: 2453 (2.51)<br><br>Length: 49 (43.0)	**Cranial US**: Normal	**Eye/vision:** Normal<br><br>**Hearing**: Normal<br><br>**Neurologic:** Microcephaly, hypotonia	No alerts in developmental screening	SGA
**37**	Second trimester<br><br>No ZIKV testing results	38 weeks<br>Female<br>Live birth	HC: 30 (0.52)<br><br>Weight: 2800 (32.0)<br><br>Length: 51(96.6)	**Cranial US**: Normal	**Eye/vision:** Not available<br><br>**Hearing**: Not available<br><br>**Neurologic:** Microcephaly	No alerts in developmental screening	No additional findings
**38**	Second trimester<br><br>No ZIKV testing results	39 weeks<br>Female<br>Live birth	HC: 32 (9.86)<br><br>Weight: 2930 (29.9)<br><br>Length: 48 (34.0)	Not available	**Eye/vision:** Visual impairment<br><br>**Hearing**: Hearing loss<br><br>**Neurologic:** Microcephaly in infant follow-up	No alerts in developmental screening	No additional findings
**39**	Second trimester<br><br>No laboratory evidence of ZIKV infection	40 weeks<br>Female<br>Live birth	HC: 31 (0.68)<br><br>Weight: 3735 (87.2)<br><br>Length: 50 (69.1)	**Prenatal US**: Normal<br><br>**Cranial US**: Normal<br><br>**Brain MRI**: Normal<br><br>**Spinal MRI:** ependymal	**Eye/vision:** Normal<br><br>**Hearing**: Normal<br><br>**Neurologic:** Microcephaly	No alerts in developmental screening	Hyperlaxity of ligaments

				tubular dilation at the level of the medullar cone, probable syringomyelia			
**40**	First trimester<br><br>Confirmed ZIKV infection	41 weeks<br>Female<br>Live birth	HC: 32 (2.56)<br><br>Weight: 3305 (43.2)<br><br>Length: 51 (79.4)	**Prenatal US**: talipes equinovarus	**Eye/vision:** Not available<br><br>**Hearing**: Not available<br><br>**Neurologic:** Microcephaly	Not available	Not available
**41**	Periconception<br><br>No ZIKV testing results	38 weeks<br>Male<br>Live birth	HC: 31 (2.06)<br><br>Weight: 2490 (6.95)<br><br>Length: 43 (0.20)	**Prenatal US:** Normal	**Eye/vision:** Bilateral strabismus<br><br>**Hearing**: Not available<br><br>**Neurologic:** Microcephaly	No alerts in developmental screening	Umbilical hernia, asymmetric ears<br><br>SGA
**42**	Second Trimester<br><br>No laboratory evidence of ZIKV infection	39 weeks<br>Female<br>Live birth	HC: 31 (3.75) (1.62)<br><br>Weight: 2747 (15.3)<br><br>Length: 49 (58.7)	**Prenatal US:** Normal<br><br>**Cranial US**: Normal	**Eye/vision:** Not available<br><br>**Hearing**: Not available<br><br>**Neurologic:** Microcephaly at birth and follow up	No alerts in developmental screening	No additional findings
**43**	Second Trimester<br><br>No ZIKV testing results	40 weeks<br>Female<br>Live birth	HC: 31 (0.68)<br><br>Weight: 2327 (0.79)<br><br>Length: 46 (2.58)	Not available	**Eye/vision:** Not available<br><br>**Hearing**: Not available<br><br>**Neurologic:** Microcephaly	Not available	SGA
**44**	Second Trimester<br><br>Confirmed ZIKV infection	40 weeks<br>Male<br>Live birth	HC: 30 (0.03)<br><br>Weight: 2655 (3.35)<br><br>Length: 50 (51.9)	**Cranial US**: Normal	**Eye/vision:** Not available<br><br>**Hearing**: Not available<br><br>**Neurologic:** Microcephaly	Not available	SGA
**45**	First trimester<br><br>Presumptive ZIKV infection	25 weeks<br>Male<br>Live birth	HC: 26 (96.22)<br><br>Weight: 940 (90.6)<br><br>Length: 34 (57.9)	**Cranial US**: Normal	**Eye/vision:** Right cataract<br><br>**Hearing**: Normal<br><br>**Neurologic:** Hypotonia	No alerts in developmental screening	Flat nasal bridge, broad forehead
**46**	First trimester<br><br>No ZIKV testing results	35 weeks<br>Male<br>Live birth	HC: 29 (1.14)<br><br>Weight: 2270 (31.1)<br><br>Length: 45 (29.4)	Not available	**Eye/vision:** Not available<br><br>**Hearing**: Not available<br><br>**Neurologic:** Microcephaly	Not available	Not available

**47**	Third trimester<br><br>Presumptive ZIKV infection	40 weeks<br>Female<br>Live birth	HC: 30 (0.07)<br><br>Weight: 2790 (10.5)<br><br>Length: 48 (21.8)	**Prenatal US:** Microcephaly<br><br>**Cranial CT:** craniofacial disproportion, reduced brain volume, overlapped sutures. Cortico-subcortical calcification in frontal lobes.<br><br>**Cranial US**: Normal	**Eye/vision:** abnormal visual evoked potentials<br><br>**Hearing**: Normal<br><br>**Neurologic:** Microcephaly, tone and movement abnormalities	Gross motor alert (12-17 months)<br><br>Hearing and language alert (12-17 months)	Varus feet, narrow forehead, high arched palate, micrognathia, hip dysplasia<br><br>SGA
**48**	First trimester<br><br>No ZIKV testing results	37 weeks<br>Female<br>Live birth	HC: not available<br><br>Weight: 2005 (1.59)<br><br>Length: 45 (8.93)	**Prenatal US:** Normal<br><br>**Cranial US**: Intracranial calcifications.<br><br>**Cranial CT**: normal ventricles, periventricular calcifications	**Eye/vision:** Normal<br><br>**Hearing**: Normal<br><br>**Neurologic:** Tone and movement abnormality	Gross motor alert (6-11 & 12-17 months)	Micrognathia, flat nasal bridge, low set ears, smooth and short philtrum, sacral dimple, hypopigmented lesions on thorax<br><br>SGA
**49**	Second trimester<br><br>No ZIKV testing results	35 weeks<br>Female<br>Live birth	HC: 44 (100)<br><br>Weight: 2820 (86.9)<br><br>Length: 43 (7.47)	**Prenatal US:** Hydrocephaly, polyhydramnios,<br><br>**Cranial US**: Absent corpus callosum, cerebral atrophy, ventriculomegaly/hydrocephaly	**Eye/vision:** Not available<br><br>**Hearing**: Not available<br><br>**Neurologic:** Tone and movement abnormalities	Not available	Cleft palate, right talipes equinovarus<br><br>Infant died at 3 days of age
**50**	First trimester<br><br>No ZIKV testing results	36 weeks<br>Male<br>Live birth	HC: 33 (64.4)<br><br>Weight: 3090 (82.5)<br><br>Length: 52 (99.3)	Not available	**Eye/vision:** Not available<br><br>**Hearing**: Not available<br><br>**Neurologic:** microcephaly in infant follow-up	No alerts in developmental screening	Bilateral hydronephrosis
**51**	First trimester<br><br>No laboratory evidence of ZIKV infection	37 weeks<br>Male<br>Live birth	HC: 29 (0.10)<br><br>Weight: 2330 (7.78)<br><br>Length: 46 (15.3)	**Prenatal US:** Ventriculomegaly, microcephaly<br><br>**Cranial CT:** Severe microcephaly, intracranial calcification, cerebral, brain	**Eye/vision:** Macular hypopigmentation, chorioretinal atrophy, vision impairment<br><br>**Hearing**: Not available<br><br>**Neurologic:** Microcephaly, seizures, tone abnormality, movement	Gross motor alert (6-11, 12-17, & 18-24 months)<br><br>Fine motor alert (6-11, 12-17, & 18-24 months)<br><br>Hearing and language alert (6-11, 12-17, & 18-24 months)	SGA

				stem, cerebellar atrophy	abnormality, , swallowing difficulty	Personal and social alert (6-11, 12-17, & 18-24 months)	
**52**	Periconception<br><br>No laboratory evidence of ZIKV infection	38 weeks<br><br>Female<br><br>Live birth	HC: 33 (49.1)<br><br>Weight: 3090 (61.6)<br><br>Length: 51 (95.5)	**Prenatal US**: microcephaly<br><br>**Cranial US**: Normal	**Eye/vision:** Normal<br><br>**Hearing**: Not available<br><br>**Neurologic:** microcephaly in infant follow-up	No alerts in developmental screening	No additional findings
**53**	Second trimester<br><br>No ZIKV testing results	40 weeks<br><br>Male<br><br>Live birth	HC: 34 (39.2)<br><br>Weight: 3380 (49.6)<br><br>Length: 53 (96.5)	Not available	**Eye/vision:** chorioretinal atrophy, vision impairment<br><br>**Hearing**: normal<br><br>**Neurologic:** microcephaly in infant follow-up	No alerts in developmental screening	No additional findings
**54**	First trimester<br><br>Presumptive ZIKV infection	38 weeks<br>Male<br>Live birth	HC: 29 (0.03)<br><br>Weight: 2450 (5.75)<br><br>Length: 47 (17.6)	**Cranial CT:** Microcephaly, ventriculomegaly, cortical atrophy, corpus callosum abnormality, large cisterna magna, abnormal cortical gyral patterns<br><br>**Brain MRI:** Ventriculomegaly, cerebral atrophy, lissencephaly, thin corpus callosum, prominent cisterna magna	**Eye/vision:** Abnormal pigment epithelium. Vision impairment.<br><br>**Hearing**: hearing loss<br><br>**Neurologic:** Microcephaly at birth and follow-up, seizures, increased body tone, swallowing difficulty, movement abnormality	Gross motor alert (6-11 & 18-24 months)<br><br>Fine motor alert (0-5, 6-11, & 18-24 months)<br><br>Hearing and language alert (6-11 & 18-24 months)<br><br>Personal and social alert (6-11 & 18-24 months)	SGA
**55**	Third trimester<br><br>No ZIKV testing results	37 weeks<br>Male<br>Live birth	HC: 29 (0.10)<br><br>Weight: 2380 (9.73)<br><br>Length: 48 (54.0)	**Prenatal US:** Microcephaly, intracranial calcifications, ventriculomegaly<br><br>**Cranial CT**: Microcephaly, multiple intracranial calcifications, ventriculomegaly, undergyrification of cortex<br><br>**Brain MRI**: Pachygyria, cerebral atrophy,	**Eye/vision:** Abnormal retinal pigment epithelium. Vision impairment<br><br>**Hearing**: Normal<br><br>**Neurologic:** Microcephaly at birth and in infant follow-up, swallowing difficulty, tone abnormality	Gross motor alert (0-5, 6-11, 12-17, & 18-24 months)<br><br>Fine motor alert (6-11, 12-17, & 18-24 months)<br><br>Hearing and language alert (6-11 & 18-24 months)<br><br>Personal and social alert (6-11 & 18-24 months)	SGA

				dysgenesis of corpus callosum, ex vacuo hydrocephaly			
**56**	First trimester<br><br>No ZIKV testing results	38 weeks<br><br>Male<br><br>Livebirth	HC: 35 (89.4)<br><br>Weight: 3280 (68.9)<br><br>Length: 52 (97.0)	**Cranial US:** Normal<br><br>**Brain MRI:** Ventriculomegaly	**Eye/vision:** Normal<br><br>**Hearing:** Normal<br><br>**Neurologic:** Normal	Hearing and language alert (0-5 & 18-24 months)	No additional findings
**57**	First trimester<br><br>No ZIKV testing results	39 weeks<br><br>Female<br><br>Livebirth	HC: 32 (9.86)<br><br>Weight: 2249 (1.05)<br><br>Length: 47 (15.3)	**Cranial US:** Normal	**Eye/vision:** Abnormal retinal pigment epithelium. visual impairment<br><br>**Hearing: normal**<br><br>**Neurologic:** microcephaly in infant follow-up	No alerts in developmental screening	SGA<br><br>Craniofacial disproportion<br><br>High arched palate<br><br>Low set ears Micrognathia<br><br>Redundant scalp skin
**58**	First trimester<br><br>No laboratory evidence of ZIKV infection	40 weeks<br>Male<br>Live birth	HC: 32 (2.27)<br><br>Weight: 2800 (6.88)<br><br>Length: 48 (11.9)	Not available	**Eye/vision:** Not available<br><br>**Hearing**: Not available<br><br>**Neurologic:** Microcephaly	Not available	SGA
**59**	First trimester<br><br>No laboratory evidence of ZIKV infection	36 weeks<br>Male<br>Live birth	HC: 35 (96.91)<br><br>Weight: 2900 (69.2)<br><br>Length: 50 (94.4)	**Cranial US**: Normal findings<br><br>**Cranial CT**: Increased sub-arachnoid space at the level of the frontal lobes.<br><br>**Brain MRI**: Frontal and parietal cortical atrophy	**Eye/vision:** Normal<br><br>**Hearing**: Hearing loss<br><br>**Neurologic:** Seizures, body tone abnormality, movement abnormality	Gross motor alert (6-11 months)<br><br>Fine motor alert (6-11 months)<br><br>Hearing and language alert (0-5 months)	Bilateral coxa valga<br><br>Broad forehead
**60**	Periconception<br><br>No ZIKV testing results	34 weeks<br>Male<br>Live birth	HC: 31 (36.1)<br><br>Weight: 1610 (6.4)<br><br>Length: 42 (7.24)	Not available	**Eye/vision:** Normal<br><br>**Hearing**: Not available<br><br>**Neurologic:** microcephaly in infant follow-up	No alerts in developmental screening	SGA
**61**	First trimester<br><br>No laboratory evidence of ZIKV infection	39 weeks<br>Male<br>Live birth	HC: 35 (82.3)<br><br>Weight: 3640 (82.8)<br><br>Length: 51 (84.6)	Not available	**Eye/vision:** Not available<br><br>**Hearing**: Not available<br><br>**Neurologic:** microcephaly in infant follow-up	No alerts in developmental screening	No additional findings

**62**	First trimester<br><br>No ZIKV testing results	38 weeks<br>Male<br>Live birth	HC: 33 (34.5)<br><br>Weight: 2900 (32.9)<br><br>Length: 55 (99.9)	**Cranial US**: Parenchymal ischemic lesions<br><br>**Cranial CT**: Vascular lesion that could be hemorrhage or ischemia in the midline<br><br>**Brain MRI:** Periventricular leukomalacia	**Eye/vision:** Abnormal retinal pigment epithelium Pale optic nerves,<br><br>**Hearing**: Normal<br><br>**Neurologic:** Microcephaly in infant follow-up, tone and movement abnormality	Gross motor alert (6-11 & 12-17 months)<br><br>Fine motor alert (6-11, 12-17, & >24 months)<br><br>Hearing and language alert (6-11, 12-17, & >24 months)<br><br>Personal and social alert (6-11, 12-17, & >24 months)	No additional findings
**63**	First trimester<br><br>No ZIKV testing results	39 weeks<br>Female<br>Live birth	HC: 34 (70.2)<br><br>Weight: 2730 (14.2)<br><br>Length: 50 (80.4)	Not available	**Eye/vision:** Normal<br><br>**Hearing**: Normal<br><br>**Neurologic:** Microcephaly in infant follow-up	No alerts in developmental screening	No additional findings
**64**	First trimester<br><br>No ZIKV testing results	38 weeks<br>Male<br>Live birth	HC: 31 (2.06)<br><br>Weight: 2650 (13.9)<br><br>Length: 50 (79.4)	**Cranial US**: Normal	**Eye/vision:** Not available<br><br>**Hearing**: Not available<br><br>**Neurologic:** Microcephaly at birth	No alerts in developmental screening	Not available
**65**	First trimester<br><br>No ZIKV testing results	37 weeks<br>Female<br>Live birth	HC: 32 (29.88)<br><br>Weight: 2700 (39.5)<br><br>Length: 47 (43.1)	**Cranial US**: No visualization of the corpus callosum, large occipital horns<br><br>**Cranial CT**: Intracranial calcifications, mild ventriculomegaly	**Eye/vision:** Strabismus<br><br>**Hearing**: Normal<br><br>**Neurologic:** microcephaly in infant follow-up, tone abnormality, movement abnormality, swallowing difficulty	Gross motor alert (6-11, 12-17, & 18-24 months)<br><br>Fine motor alert (6-11, 12-17, & 18-24 months)<br><br>Hearing and language alert (6-11, 12-17, & 18-24 months)<br><br>Personal and social alert (6-11, 12-17, & 18-24 months)	Nasal deviation to the left
**66**	Second Trimester<br><br>No ZIKV testing results	39 weeks<br>Female<br>Live birth	HC: 35 (91.68)<br><br>Weight: 3000 (36.8)<br><br>Length: 48 (34.0)	**Cranial US**: Normal	**Eye/vision:** Left enophthalmos, right congenital cataract, presumptive staphyloma<br><br>**Hearing:** Not available<br><br>**Neurologic**: Not available	Not available	Persistent ductus arteriosus, anterior anus, enlarged clitoris, low set ears<br><br>Infant death at 5 months of age
**67**	First trimester<br><br>No ZIKV testing results	33 weeks<br>Male<br>Live birth	HC: 25 (0.01)<br><br>Weight: 980 (1.00)<br><br>Length: 35 (0.03)	**Prenatal US**: Suspected aneuploidy, microcephaly, polyhydramnios	**Eye/vision:** Not available<br><br>**Hearing**: Not available<br><br>**Neurologic:** Microcephaly, congenital contractures, bilateral talipes equinovarus	Not available	SGA<br><br>Infant died 1 minute after birth

*Percentiles for weight, length and head circumference at birth based on INTERGROWTH 21st standards (32)
†Brain anomaly described as holoprosencephaly, but insufficient information from imaging or clinical examination to confirm diagnosis.
SGA, small for gestational age (defined as <10th percentile of birth weight by gestational age and sex using INTERGROWTH 21st standards) (32)

Table S3. Characteristics among pregnant women with complete pregnancy outcome data, comparing those with any Zika virus laboratory testing results, and those with no Zika virus laboratory testing results, Project *Vigilancia de Embarazadas con Zika* (VEZ), Colombia, 2016 – 2018.

	**Total (N = 1180) N (%)**	**Pregnant women with testing results (N =569) N (%)**	**Pregnant women without testing results (N = 611) N (%)**	**Chi-Sq p-value**
**Zika virus laboratory testing**				
Positive	254 (21.5)	254 (44.6)	Not applicable	Not applicable
Confirmed*	220 (86.6)	220 (86.6)	Not applicable	
Presumptive*	34 (13.4)	34 (13.4)	Not applicable	
Negative	315 (26.7)	315 (55.4)	Not applicable	
**Maternal age at conception (years)**				0.1830
<17	106 (9.0)	51 (9.0)	55 (9.0)	
17-19	208 (17.6)	93 (16.3)	115 (18.8)	
20-24	374 (31.7)	181 (31.8)	193 (31.6)	
25-29	249 (21.1)	120 (21.1)	129 (21.1)	
30-34	149 (12.6)	76 (13.4)	73 (12.0)	
>=35	78 (6.6)	35 (6.2)	43 (7.0)	
Not reported	16 (1.4)	13 (2.3)	3 (0.5)	
**Maternal education**				0.0002
< Secondary	120 (10.2)	60 (10.5)	60 (9.8)	
Secondary	595 (50.4)	269 (47.3)	326 (53.4)	
Technical	130 (11.0)	48 (8.4)	82 (13.4)	
Professional	70 (5.9)	35 (6.2)	35 (5.7)	
Not reported	265 (22.5)	157 (27.6)	108 (17.7)	
**Gravidity**				<0.0001
Primigravid	323 (27.4)	190 (33.4)	133 (21.8)	
Multigravid	668 (56.6)	298 (52.4)	370 (60.6)	
**Delivery method**				0.0204
Vaginal	541 (45.9)	282 (49.6)	259 (42.4)	
Caesarean section	622 (52.7)	282 (49.6)	340 (55.7)	

Not reported	17 (1.4)	5 (0.9)	12 (2.0)	
**Infant sex****				0.2728
Male	576 (48.1)	273 (47.5)	303 (48.7)	
Female	603 (50.4)	290 (50.4)	313 (50.3)	
Not reported	18 (1.5)	12 (2.1)	6 (1.0)	
**Plurality**				0.1715
Singleton	1150 (97.5)	553 (97.2)	597 (97.7)	
Twins	12 (1.0)	4 (0.7)	8 (1.3)	
Not reported	18 (1.5)	12 (2.1)	6 (1.0)	
**Symptoms reported‡**				
Rash	983 (83.3)	471 (82.8)	512 (83.8)	0.6387
Fever	787 (66.7)	358 (62.9)	429 (70.2)	0.0079
Joint pain	691 (58.6)	336 (59.1)	355 (58.1)	0.7408
Headache	612 (51.9)	277 (48.7)	335 (54.8)	0.0347
Conjunctivitis	267 (22.6)	124 (21.8)	143 (23.4)	0.5085
Eye pain	116 (9.8)	58 (10.2)	58 (9.5)	0.6862
Diarrhea	99 (8.4)	50 (8.8)	49 (8.0)	0.6411
Swollen lymph nodes	22 (1.9)	11 (1.9)	11 (1.8)	0.8661
Not reported	72 (6.1)	39 (6.9)	33 (5.4)	0.2974
**Trimester of symptom onset**				0.0281
Periconception (up to 8 weeks before estimated date of conception)	84 (7.1)	30 (5.3)	54 (8.8)	
First trimester	616 (52.2)	314 (55.2)	302 (49.4)	
Second trimester	397 (33.6)	189 (33.2)	208 (34.0)	
Third trimester	63 (5.3)	24 (4.2)	39 (6.4)	
Not reported	20 (1.7)	12 (2.1)	8 (1.3)	

*Confirmed: Zika virus nucleic acid amplification testing (NAAT) of maternal serum or urine, amniotic fluid, umbilical cord serum or cord tissue, placental tissue, or fetal tissue was positive, regardless of Zika virus immunoglobulin M (IgM) test results; Presumptive: Zika virus NAAT of maternal serum or urine, amniotic fluid, umbilical cord serum or cord tissue, placental tissue, or fetal tissue was negative or not performed, but maternal or fetal Zika virus IgM test results were positive and dengue virus IgM test results were negative. Of note, the interval between symptom onset and NAAT was often prolonged.

** Overall denominator (n=1,192) includes 1,178 live born infants and 14 pregnancy losses. Denominator for subgroup with laboratory testing results (n=573) includes 564 live born infants and 9 pregnancy losses. Denominator for subgroup without laboratory testing results (n=619) includes 614 live born infants and 5 pregnancy losses

‡ Not mutually exclusive; participants could report more than one symptom.

Table S4. Zika virus laboratory testing results, and documentation of samples among those with no Zika virus laboratory testing results, Project *Vigilancia de Embarazadas con Zika* (VEZ), Colombia, 2016 – 2018.

**Pregnant women with Zika virus laboratory testing results (N = 569/1180; 48.2%)**	
	**n (%)**
Positive	254 (44.6)
Confirmed*	220 (86.6)
Presumptive*	34 (13.4)
Negative	315 (55.4)
**Pregnant women without Zika virus laboratory testing results (N = 611/1,180; 51.8%)**	
	**n (%)**
Documentation of sample collected during pregnancy	597 (97.7)
Sample collected as part of Project VEZ	184 (30.8)
Documentation of sample collected as part of national surveillance (Sivigila) as noted in medical record	413 (69.2)
No documentation of sample collected during pregnancy	14 (2.3)

*Confirmed: Zika virus nucleic acid amplification testing (NAAT) of maternal serum or urine, amniotic fluid, umbilical cord serum or cord tissue, placental tissue, or fetal tissue was positive, regardless of Zika virus immunoglobulin M (IgM) test results; Presumptive: Zika virus NAAT of maternal serum or urine, amniotic fluid, umbilical cord serum or cord tissue, placental tissue, or fetal tissue was negative or not performed, but maternal or fetal Zika virus IgM test results were positive and dengue virus IgM test results were negative. Of note, the interval between symptom onset and NAAT was often prolonged.
